# Study of cytokine-induced immunity in bullous pemphigoid: recent developments

**DOI:** 10.1080/07853890.2023.2280991

**Published:** 2023-12-18

**Authors:** Ruiting Huang, Lingyu Hu, Fuqiong Jiang

**Affiliations:** Department of Dermatology, The Second Affiliated Hospital of Kunming Medical University, Kunming, China

**Keywords:** Bullous pemphigoid, cytokines, interferon-γ, interleukin-4, Th1 cells, Th2 cells, Th17 cells

## Abstract

Bullous pemphigoid (BP) is an organ-specific disease. Its pathogenesis has not been clearly studied yet; However, studies in recent years have shown that its pathogenesis is related to T helper cells. The pathogenesis of BP is mainly related to Th2 and Th17-related cytokines. IL-4, IL-5 and IL-13 cause eosinophil recruitment, promote antibody production, trigger pruritus and promote blister formation and other symptoms. IL-17 and IL-23 promote the production of matrix metalloproteinase-9 (MMP-9) by related cells, which causes dermo-epidermal junction (DEJ) separation to form bullae and blisters, and can persist in BP inflammation. The serum concentrations of IL-17 and IL-23 are related to the prognosis of BP. In this paper, we focus on the role of related cytokines in the pathogenesis of bullous pemphigoid and the relationship between the related cytokine populations secreted by three major T helper cells—helper T lymphocytes 1 (Th1), Th2, and Th17. A better understanding of the biological and immunological functions of cytokines associated with BP patients will provide opportunities for therapeutic targets in BP.

## 1. Background

Bullous pemphigoid (BP) is an autoimmune subepidermal bullous disease caused by the production of antibodies targeting cell adhesion molecules. These antibodies can disrupt the connection between the epidermis and the basement membrane, resulting in the formation of vesicles and bullae. In recent years, researchers have categorized the categories of inflammatory response according to the distinct T helper (Th) cells differentiated by stimulation of primitive T cells and the secreted cytokine populations [1]. Th1 cells and their secreted cellular inflammatory factors are primarily responsible for the type 1 inflammatory response. The type 2 inflammatory response is characterized by an immune inflammatory response produced mainly by Th2 cells, type 2 innate lymphoid cells, and their secreted cytokine populations [2]. Th17 cells and their secreted cytosolic inflammatory factors are primarily responsible for the type 3 inflammatory response [3]. Numerous studies have demonstrated that Th cells play a significant role in the pathogenesis of BP, and an increasing number of researchers are investigating the cytokines associated with BP pathogenesis [4,5]. To explore the relationship between different classes of cytokines and BP, we reviewed the literature on three main classes of T lymphocyte-associated cytokines involved in these three types of inflammatory responses.

## 2. Th1-like cytokines associated with BP

Th1 cells and the cytosolic inflammatory factors they secrete are mainly responsible for the type 1 inflammatory response. Th1 cells primarily secrete interleukin-2 (IL-2), interleukin-12 (IL-12), interferon-γ (IFN-γ) and tumour necrosis factor β (TNF-β), and mediate immune responses related to cytotoxicity and local inflammation. They also participate in cellular immunity and the progression of delayed hypersensitivity associated with diseases such as multiple sclerosis, autoimmune type 1 diabetes and rheumatoid arthritis. They also play an important role in the prevention of infection by intracellular pathogens (viruses, bacteria and parasites) [6].

The BP180 non-collagenous 16A (BP180NC16A) domain is the major immunodominant region, and T cells from patients with BP exhibit specific proliferative responses to recombinant forms of the BP180NC16A domain. They proliferate *in vitro* with specific BP180-reactive CD4^+^ T cells that are cloned with CD4^+^ T cells and produce Th1- and Th2-associated cytokines [7]. Using reverse PCR and specific cytokine primers, it has been observed that BP180-specific T cell lines produce IL-2, IL-4, IL-5, IL-6 and IFN-γ at the mRNA level as well as at the protein level [8]. These studies indicate the involvement of self-reactive Th1 and Th2 cells in the pathogenesis of BP.

The characteristic chemokine receptors of Th1 cells are CXCR3 and CCR5 [9]. The ligands of CXCR3 are CXCL9, CXCL10 and CXCL11, and the ligands of CCR5 are CCL2, CCL3, CCL4 and CCL5, which primarily contribute to the recruitment of Th1 cells. Studies have shown that patients with BP have significantly elevated serum CXCL10 levels [10], and it has also been reported that patients with BP have significantly elevated serum CCL2 and CXCL10 levels. This correlates positively with disease severity [11]. However, the source of these chemokines is unknown.

In contrast, BP180 could produce Th1 cell responses in healthy controls, and peripheral T cells that reacted with BP180 were detected in healthy donors using an enzyme-linked immunospot assay. These T cells predominantly belonged to the Th1 subpopulation, which produces IFN-γ [12]. Therefore, the role of Th1-related cytokines in the pathogenesis of BP is controversial.

## 3. Th2-like cytokines associated with BP

The type 2 inflammatory response is mostly linked to Th2 cells, type 2 innate lymphoid cells and the number of cytokines that they release. The primary function of type 2 inflammatory cytokines is to stimulate the proliferation of B cells and to produce antibodies that are associated with humoral immunity, and they also play an important role in allergic and atopic diseases [13,14]. They are also involved in barrier immunity on mucosal surfaces and in protection against parasitic infections [15].

Although a mixed spectrum of Th1 and Th2 was initially thought to be the main mediator of the immune response to BP, as studies progressed, it was discovered that there was a significant infiltration of inflammatory cells, primarily eosinophils and CD4^+^ T cells, in the upper dermis of patients with BP, in addition to the presence of large numbers of eosinophils and their activated cytokines and chemokines in the serum [16,17]. Further research revealed that Th2-related cytokines and chemokines, such as IL-4, IL-5, CCL-13, CCL-18 and the eosinophil chemokine (eotaxin), were highly expressed in BP lesions [18]. The titer of CCL-18 in the serum of patients with BP was 1.84 times higher than that of healthy controls, and its titer was linearly correlated with the patients’ clinical scores. The titer of CCL-18 in the patients’ blister fluid was even higher, at five times the titer of their own serum [19], indicating that the pathogenesis in patients with BP was primarily mediated by Th2 inflammation. In addition, clones of T cell lines responsive to specific peptides in the BP180 antigen have been identified in patients with BP, and these BP180-specific Th cells characteristically secrete Th2 cytokines, which also contribute to the recruitment of eosinophils.

In conclusion, this advantage of Th2 cytokines is indicative of BP. Specifically, the Th2-like cytokines associated with BP consist primarily of IL-4, IL-5, IL-10, IL-13 and IL-15.

IL-4 is an essential regulator of Th2 cell differentiation and a crucial factor in the B cell isotype switch. It is primarily linked to the production and activation of B cells, which enables them to produce autoantibodies [20]. Experiments revealed that stimulation of peripheral blood mononuclear cells (PBMCs) with recombinant BP180NC16A domain significantly increased IL-4 secretion [21]. In addition, 22 overlapping peptides spanning the entire sequence of the BP180 NC16A domain were synthesized in experiments, and the sensitivity of Th2 cells from patients with BP to these peptides was investigated using ELISPOT. It was demonstrated that two major epitope peptides, P2 (492-506 aa: VRKLKARVDELERIR) and P5 (501-515 aa: ELERIRRSIL PYGDS), stimulated CD4^+^ T cell proliferation by inducing the production of IL-4 cytokines and the activation of B cell autoantibody secretion [22].

IL-5 is primarily associated with eosinophil recruitment and activation, and it has been demonstrated that IL-4 and IL-5 enable eosinophils in patients with BP to respond to blister fluid at lower concentrations [23,24]. At the same time, IL-5 can also induce the separation of the dermo-epidermal junction (DEJ) through matrix metalloproteinase-9 (MMP-9) production. In the mouse model, MMP-9 can influence downstream neutrophil elastase activity by deactivating α1-protease inhibitor, resulting in the degradation of BP180 and the eventual separation of DEJ [25]. In addition, IL-5 levels correlate with BP disease activity. A meta-analysis revealed that the IL-5 level in serum and vesicles was significantly elevated in patients with BP [26].

The frequency of regulatory B cells producing the anti-inflammatory factor IL-10 was not significantly different when stimulating PBMC in Kabuto’s study, after the number of PBMC in the healthy control and BP groups were controlled [27]. Other studies have demonstrated that the IL-10 level in the serum of patients with BP before treatment was comparable to that of healthy controls, but increased in patients with BP after treatment [28]. The increase in the number of cells producing IL-10 as a result of treatment with corticosteroids may account for the elevated IL-10 serum levels. In contrast, patients with severe clinical manifestations and treatment resistance were frequently associated with low levels of IL-10, which regulates T-cell secretion [29]. Alternatively, elevated levels of IL-10 were only detected in the serum of patients who achieved complete remission when treated with rituximab. In conclusion, IL-10 may be relatively deficient in the serum of patients with BP and cannot assure adequate immunoregulation in states of enhanced immune response; however, the therapeutic application of IL-10 is associated with improvement in the treatment of BP [30,31].

The IL-13 level in the serum of patients with BP was significantly higher than that of the healthy subject group. Wang et al. found that IL-13 was associated with BP in terms of allele, genotype, haplotype and serum cytokine concentration [32]. The up-regulated expression of IL-13 occurs primarily through the induction of endothelial vascular cell adhesion molecule-1 (VCAM-1) to bind to the integrin α4β1 receptor on relevant inflammatory cells, resulting in the migration of cells such as eosinophils, macrophages and T cells [33]. Serum immunoglobulin E (IgE) is elevated in patients with BP. It has been reported that 22–100% of patients with BP have elevated serum IgE levels and IgE titers are correlated with disease activity [34,35]. IL-13 can facilitate the differentiation of B cells into mature plasma cells and mediate the plasma cells secretion of IgE antibodies [32, 36]. This indicates that IL-13 is related to the pathogenesis of BP.

In the blister fluid of patients with BP, a significantly positive correlation between IL-15 and IL-5 concentrations has been found, as well as a positive correlation between IL-15 and lesion severity [37]. As an upstream cytokine, IL-15 can bind to the IL-15 receptor on helper T cells to activate the signal transducer and activator of transcription 5 (STAT5) and produce IL-5, IL-13 and eosinophil chemotactic protein (eotaxin) [38,39]. In fact, the IL-15 gene is the most frequently expressed cytokine in B cells specific for BP180. Therefore, IL-15 may be a cytokine that plays a crucial role in the eosinophilia found in patients with BP.

In conclusion, the activation of Th2 cells and the production of related cytokines can further stimulate the proliferation of B cells and the production of autoantibodies, promoting the transition of immunoglobulins to IgG4 and IgE subtypes, mast cell degranulation and eosinophil activation. In turn, eosinophils promote the differentiation of T cells into Th2 cells, thereby generating positive feedback that maintains the hyperinflammatory state in patients with BP.

## 4. Th17-like cytokines associated with BP

Th17 cells and their secreted IL-17, IL-22 and IL-23 cellular inflammatory factors are primarily involved in type 3 inflammatory response and can prevent infections through the recruitment of neutrophils and macrophages [40]. They are associated with anti-extracellular bacterial and antifungal infections, as well as playing a role in psoriasis and inflammatory bowel disease, and have been studied in patients with BP [41].

Immunohistochemical staining demonstrated the presence of Th17 cells in the lesions of patients with BP. Under certain conditions, myeloid dendritic cells (mDC) can produce IL-1β and IL-23, which can mediate the maturation of Th17 from naive cluster differentiated antigen 161 (CD161)-positive T cells and induce Th17 to produce a series of cytokines represented by IL-17 [42]. Studies have been conducted to treat wild-type mice injected with anti-BP180 IgG with anti-IL-17A and an isotype-control antibody. They demonstrated that mice treated with anti-IL-17A antibodies had significantly fewer lesions than mice treated with a control antibody [43]. According to a number of studies, IL-17 and IL-23 can stimulate monocytes and neutrophils to produce MMP-9 [44,45]. The protease can mediate the dermo-epidermal division. The effect of IL-17 on MMP-9 secretion was prominent, despite the fact that IL-17 levels in the serum of patients with BP did not differ from those of the healthy group and were unrelated to disease severity. In addition, the serum levels of IL-17 and IL-23 remained elevated in patients with BP at risk for relapse. Meanwhile, both IL-17 and IL-23 independently induce the formation of extracellular traps (ETs) in patients with BP relapse, particularly neutrophil ETs, which are surrounded by chromatin DNA filaments and granule proteins as they are released by neutrophils to trap microbiomes. In patients with BP, they can be precisely located at the edge of the dermo-epidermal separation. *In vitro* experiments have also demonstrated that the degradation of DNA filaments inhibits the division of DEJ [46]. In conclusion, IL-17 is associated with vesicle formation in patients with BP.

In addition, the serum concentration of IL-17 can influence the therapeutic effects of glucocorticoids. Steroid resistance is associated with increased expression of glucocorticoid receptor β (GR-β) [47,48], and IL-17 and IL-23 can significantly upregulate the expression of GR-β mRNA, thereby affecting the response to BP treatment [49].

## 5. Conclusions

In conclusion, the pathogenesis of BP is primarily associated with Th2 and Th17-related cytokines, which cause eosinophil recruitment by IL-4, IL-5 and IL-13, promote antibody production, trigger pruritus, and promote vesicle formation and other symptoms, while IL-17 is also associated with the formation of bullae and vesicles that can perpetuate inflammation in BP. The serum concentration is associated with disease progression.

Some biologics, such as dupilumab, that target the above cytokines can inhibit the IL-4/13 signal pathway in a targeted manner and have been used clinically to induce therapeutic effects in a variety of diseases with type 2 inflammatory response. Currently, a multicentre, randomized, double-blind, parallel-group, placebo-controlled clinical trial (NCT04206553) is investigating dupilumab efficacy in BP. The efficacy is adequate based on the number of individual cases reported [50]. Of course, some failures were also reported, such as the phase II clinical trial of an IL-5-targeting drug for BP, which ultimately ended in failure [51].

**Figure 1.**
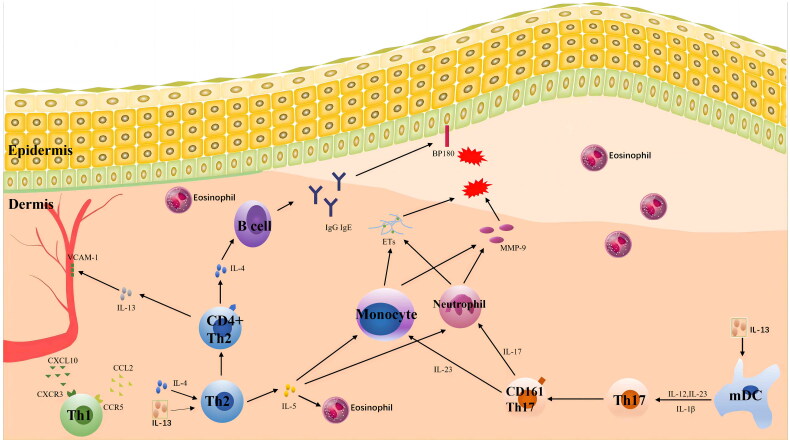
A network of cytokines affects the pathogenesis of BP. The pathogenesis of BP is primarily associated with cytokines related to Th2 and Th17. IL-4, IL-5 and IL-13 cause the recruitment of eosinophils, stimulate the production of antibodies, induce pruritus, promote blister formation and cause other symptoms. IL-17 and IL-23 stimulate the production of matrix metalloproteinase-9 (MMP-9) by related cells, which leads to dermo-epidermal junction (DEJ) separation and the formation of bullae and blisters and can persist in BP inflammation. The serum concentrations of IL-17 and IL-23 are associated with the prognosis of BP.

In conclusion, additional research is required to ascertain how a large and complex network of cytokines affects the pathogenesis of BP (Figure 1), as well as the use of immunoregulatory and clinical biological preparations.

## Data Availability

The datasets used and/or analysed during the current study available from the corresponding author on reasonable request.
